# The velocity of collateral filling predicts recanalization in acute ischemic stroke after intravenous thrombolysis

**DOI:** 10.1038/srep27880

**Published:** 2016-06-14

**Authors:** Sheng Zhang, Xiaocheng Zhang, Shenqiang Yan, Yangxiao Lai, Quan Han, Jianzhong Sun, Minming Zhang, Mark W. Parsons, Shaoshi Wang, Min Lou

**Affiliations:** 1 Department of Neurology, the Second Affiliated Hospital of Zhejiang University, School of Medicine, Hangzhou, China; 2 Department of Radiology, the Second Affiliated Hospital of Zhejiang University, School of Medicine, Hangzhou, China; 3 Department of Neurology, John Hunter Hospital, The University of Newcastle, Newcastle, New South Wales, Australia; 4 Department of Neurology, Shanghai Jiaotong University Affiliated Branch of People’s No. 1 Hospital, Shanghai, China

## Abstract

The aim of this study was to evaluate the impact of pretreatment quality of collaterals, involving velocity and extent of collateral filling, on recanalization after intravenous thrombolysis (IVT). A retrospective analysis was performed of 66 patients with acute middle cerebral artery (MCA) M1 segment occlusion who underwent MR perfusion (MRP) imaging before IVT. The velocity of collateral filling was defined as arrival time delay (ATD) of contrast bolus to Sylvian fissure between the normal and the affected hemisphere. The extent of collateral filling was assessed according to the Alberta Stroke Program Early CT (ASPECT) score on temporally fused maximum intensity projections (tMIP). Arterial occlusive lesion (AOL) score was used to assess the degree of arterial recanalization. ATD (OR = 0.775, 95% CI = 0.626–0.960, *p* = 0.020), but not tMIP-ASPECT score (OR = 1.073, 95% CI = 0.820–1.405, *p* = 0.607), was independently associated with recanalization (AOL score of 2 and 3) at 24 hours after IVT. When recanalization was achieved, hemorrhagic transformation (HT) occurred more frequently in patients with slow collaterals (ATD ≥ 2.3 seconds) than those with rapid collaterals (ATD < 2.3 seconds) (88.9% vs 38.1%, *p* = 0.011). In conclusion, the velocity of collaterals related to recanalization, which may guide the decision-making of revascularization therapy in acute ischemic stroke.

Recanalization is the aim of intravenous thrombolysis (IVT) in acute proximal intracranial arterial occlusion, in order to attenuate penumbra loss^1^. Large amount of evidences revealed that leptomeningeal collaterals (LMCs) may sustain the ischemic penumbra prior to recanalization and impact the rate of recanalization after revascularization therapy^2^,^3^,^4^. Pretreatment assessment of LMCs is therefore critical to refine therapeutic decision-making in patients with acute occlusion of major cerebral arteries.

Noninvasive assessment of LMCs is ideal since it quickly provides more information of collaterals by including contralateral view, anterior and poster view simultaneously, as opposed to the conventional angiographic evaluation. Recently, a CT-based collateral assessment, for the first time, was demonstrated to predict clinical response to endovascular therapy in the Endovascular Treatment for Small Core and Anterior Circulation Proximal Occlusion With Emphasis on Minimizing CT to Recanalization Times (ESCAPE) trial^5^. However, no MR-based collateral assessment has been prospectively applied in patient selection for revascularization therapy, although retrospective studies have observed good correlation between MR and conventional angiography-based collateral grading systems^6^,^7^.

Moreover, comprehensive assessments which mixed the temporal and morphological information of LMCs was widely used, while the evidence regarding the value of each aspect of collaterals for the response to IVT is still limited. The effect of velocity and extent of collateral filling on recanalization may be different. Olindo *et al*. found that the extent of fluid-attenuated inversion recovery (FLAIR) vascular hyperintensities (FVHs) that characterized the territory of slow retrograde collateral blood supply in acute ischemic stroke, showed no association with recanalization after IVT^8^. Conversely, Nicoli *et al*. discovered that MR perfusion parameter – *T*max was associated with collateral status and recanalization^3^. In the setting of major cerebral arterial occlusion, *T*max reflects the time delay of cerebral perfusion via collateral routes, which implicates the connection between collateral filling velocity and recanalization. However, no evidence has been provided as to the respective relationship between filling velocity, morphologic extent of collaterals and recanalization in patients with acute large arterial occlusion.

Therefore, in this study, we sought to use MR perfusion (MRP) data to develop a novel method of evaluating the velocity and extent of collaterals, as well as to determine their associations with recanalization after IVT in patients with proximal intracranial arterial occlusions.

## Methods

### Ethics Statement

Written informed consent was obtained from each patient or an appropriate family member. The human ethics committee of The Second Affiliated Hospital of Zhejiang University approved the protocol of this study. All clinical investigations were conducted according to the principles expressed in the Declaration of Helsinki.

### Patient selection

We retrospectively reviewed our prospectively collected database for consecutive patients with acute ischemic stroke who received intravenous thrombolysis (IVT) between June 2009 and June 2015. We then included patients who (i) received IVT within 6 hours of symptom onset; (ii) had a complete occlusion of middle cerebral artery (MCA) M1 segment, with or without an occlusion of the internal carotid artery (ICA) on baseline time-of-flight magnetic resonance angiography (TOF-MRA); (iii) had baseline diffusion-weighted imaging (DWI), perfusion-weighted imaging (PWI), and susceptibility-weighted imaging (SWI); (iv) underwent 24 hours follow-up TOF-MRA; (v) underwent SWI or non-contrast CT at 24 hours after IVT; (vi) prestroke modified Rankin Scale (mRS) score ≤ 2. Patients were excluded if: i) antegrade flow across incomplete occlusion; ii) poor-quality MR imaging due to motion artifacts. Intravenous rt-PA was administered according to the international guidelines (0.9 mg/kg, 90 mg dose at maximum, 10% in a bolus in 1 minute with the remaining dose in a 60-minutes infusion).

### Imaging acquisition

Baseline multi-modal MRI was performed on a 3.0 T system (Signa Excite HD, General Electric Medical system, Milwaukee, USA) that equipped with an 8-channel phased array head coil. Foam pads were inserted into the space between the subject’s head and the MRI head coil to minimize head motion. The MRI protocol included an axial isotropic DWI (field of view = 240 mm, slice thickness = 1 mm, number of slices = 18, slice gap = 1 mm, acquisition matrix = 160 × 160, duration = 32 seconds); TOF-MRA (TR = 20 ms; TE = 3.2 ms; flip angle = 15°; acquisition matrix = 320 × 224, section thickness = 1.4 mm, 3 slabs, duration = 3 minutes 46 seconds); SWI (TE = 4.5 ms (first echo); inter-echo spacing = 4.5 ms; TR = 58 ms; FOV = 24 × 24 cm^2^; matrix size = 256 × 256; flip angle = 20°; slice thickness = 2.0 mm with no gap between slices; duration = 3 minute 27 seconds); and perfusion-weighted imaging (PWI) (field of view = 240 mm, repetitive time = 1500 ms, echo time = 30 ms, acquisition matrix = 128 × 128. Repetitive scanning times = 50, gadolinium dose = 15 mL, contrast speed = 4–5 mL/s, duration = 1 min 15 s). The entire duration of the MR imaging protocol was 15 minutes.

### Assessment of collaterals on PWI

All admission and follow-up images were reviewed and reconstructed using commercial software (MIStar; Apollo Medical Imaging Technology, Melbourne, Australia). We subtracted PWI raw images from each frame of the raw perfusion data as in DSA, and obtained axial, sagittal and coronal reconstructions. Prior to the arterial input function (AIF) selection, images were reviewed (5 seconds per frame via a movie player tool of MIStar) by 1 stroke fellow (S.Z, with over 3 years of experience in stroke imaging) in order to confirm that the contrast visualized beyond the apparent complete occlusion on MRA was truly from retrograde filling.

#### (1) Assessment of the velocity of collateral flow

**Figure 1:**
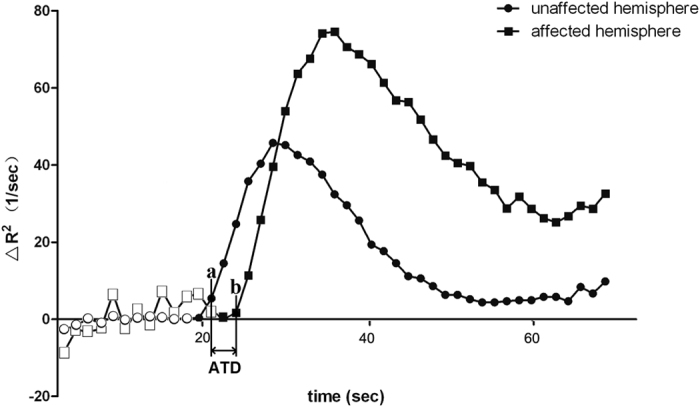
The time attenuation curves (TACs) of the ischemic and contralateral hemisphere. Hollow dots indicate time points before contrast inflow. Point a and b indicate the first time point when contrast reached the selected artery on the arterial input function (AIF) curve of the contralateral and ischemic hemisphere, respectively. Arrival time delay (ATD) was defined as the time difference between these two points. In this case, the first time point of contrast inflow of both sides were 21.86 seconds and 24.46 seconds, respectively. Thus, the ATD was 3.6 seconds.

The velocity of collateral flow was quantified on PWI-derived subtraction images. The artery acrossed the sylvian fissure and located at the end of MCA M1 segment of each hemisphere was detected to generate the AIF curve, respectively. Of note, the artery was primarily selected at the contralateral hemisphere and the region of interest (ROI) should be symmetrically placed to select the artery at the ischemic hemisphere. The first time point when contrast reached the selected artery was then identified on AIF curve of each hemisphere. The arrival time delay (ATD) was defined as the time difference between these two time points (see Fig. 1).

#### (2) Assessment of the extent of collaterals

**Figure 2:**
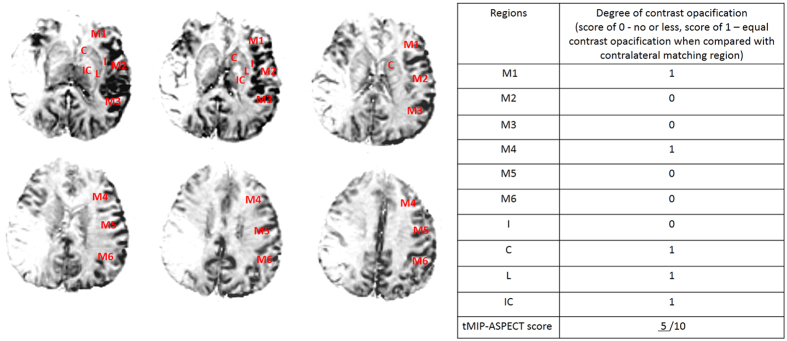
Six consecutive slices on PWI tMIP images that cover almost the whole territory of the middle cerebral artery were used to assess the extent of collateral filling. tMIP-ASPECT score is based on scoring the degree of contrast opacification (0 - no or less, 1 – equal contrast opacification compared with matching region in contralateral hemisphere) in 10 regions, including C caudate, L lentiform nucleus, IC internal capsule, I insular ribbon and M1–6 cortical regions of the middle cerebral artery (M1–3 at the level of basal ganglia, M4–6 at the level rostral to the ganglionic structures). In this patient, the deficit of collateral blood flow involved M2–3, M5–6, and I, and the tMIP-ASPECT score was 5.

Temporally fused MIP (tMIP) reconstructions^9^ from PWI images that fuse contrast opacification across the duration were obtained. Six consecutive slices (5 mm each) that cover almost the whole territory of MCA were used to assess the extent of collateral based on Alberta stroke program early CT (ASPECT) score (see Fig. 2).

Two stroke fellows who were blinded to other imaging and clinical data independently assessed ATD and tMIP–ASPECT score. Cases with difference of over 2 seconds for ATD were regraded by consensus. Bland-Altman statistics showed that difference of ATD was −0.7 (95% limits of agreement: −12.1–10.8) and tMIP–ASPECT score was 0.82 (95% limits of agreement: 1.58–0.06) between 2 raters. Inter-rater agreement between 2 raters for ATD (ICC: 0.898, 95% CI: 0.832–0.939) and tMIP–ASPECT score (ICC: 0.984, 95% CI: 0.973–0.991) were excellent.

### Radiological and clinical assessment

Stroke severity was assessed with National Institutes of Health Stroke Scale (NIHSS) serially during hospitalization and whenever neurologic worsening occurred. An arterial occlusive lesion (AOL) scale was rated on 24-hours TOF-MRA by the consensus of 2 stroke fellows (S.Z and SQ. Y), with assignments of 0 (no recanalization), 1 (incomplete or partial recanalization, with no distal flow), 2 (incomplete or partial recanalization, with any distal flow), and 3 (complete recanalization, with any distal flow)^10^. Patients were dichotomized into recanalization (AOL score, 2–3) versus non-recanalization (AOL score, 0–1). Hemorrhagic transformation (HT) was identified on 24-hours SWI images. HT was classified as hemorrhagic infarction (HI) and parenchymal hemorrhage (PH), according to the definitions used in The European Cooperative Acute Stroke Study (ECASS)^11^. Symptomatic hemorrhagic transformation (sHT) was defined as any intracranial hemorrhage associated with an increase of ≥4 points of NIHSS, or death^12^. 3-months modified Rankin Scale (mRS) score was collected, and mRS score of 0–2 was defined as good outcome. Threshold of Tmax > 6 seconds was used for volumetric measurement of baseline hypoperfusion^13^. Initial DWI lesion was used as baseline infarct volume, and 24-hours DWI or NCCT for the final infarct volume. Volumetric analysis was performed with MIStar software.

### Statistical analysis

All metric and normally distributed variables were reported as mean ± standard deviation; non-normally distributed variables as median (25^th^–75^th^ percentile). Categorical variables were presented as frequency (percentage). Comparison between groups were assessed by using student *t* test for parametric data, Mann-Whitney *U* test for nonparametric data, and Pearson Chi-Square test for categorical data. Spearman correlation coefficient was used to analyze the association of collateral status with radiological and clinical variables. Multivariate regression analyses were conducted to determine independent predictors of recanalization and infarct growth at 24 hours after IVT. Results are reported as odds ratios (ORs) with 95% confidence intervals (CIs). Receiver operating characteristic (ROC) curve analysis was performed to determine the optimal threshold. A *p* value of <0.05 was considered to be statistically significant. All statistical analyses were conducted using SPSS, Version 19.0 (IBM, Armonk, New York).

## Results

Among our cohort of 185 patients with baseline PWI, 66 patients had acute occlusion of MCA-M1 and were included for final analysis. The mean age was 69 ± 6 years old with 28 (42.4%) being women. The mean admission NIHSS was 14 (9–18), median time from onset to MR scanning was 187 (140–244) minutes, and median time from onset to treatment was 235 (180–290) minutes, respectively. Occlusion of both ICA and MCA M1 segment was found in 24 (36.4%) patients. After IVT, 30 (45.5%) patients achieved recanalization, of whom 16 (53%) patients had HT and 2 (6.7%) patients had sHT, and 17 (56.7%) had good outcome.

AOL score was significantly associated with 3-months mRS score (ρ = −0.392, p = 0.000). Patients with recanalization were more likely to have good outcome when compared with those with non-recanalization (56.7% vs 16.7%, χ^2^ = 11.532, p = 0.001).

Univariate analysis showed that ATD was strongly associated with tMIP-ASPECT score (ρ = −0.430, *p* = 0.000). Both ATD and tMIP-ASPECT score were associated with baseline NIHSS score, baseline hypoperfusion and infarct volume, and final infarct volume (all *p* < 0.05). However, only ATD (ρ = −0.264, *p* = 0.032), but not tMIP-ASPECT score (ρ = 0.153, *p* = 0.221), was associated with AOL score.

As illustrated in Table 1, univariate analysis showed that patients with recanalization had shorter ATD (1.67 ± 2.29 vs 4.13 ± 4.88 seconds, Z = −2.653, *p* = 0.008) and less rate of both ICA and MCA-M1 occlusion in comparison of patients with non-recanalization, while tMIP-ASPECT score was not significantly different between these two groups. Multivariate logistic regression analysis identified that ATD (OR = 0.775, 95% CI = 0.626–0.960, *p* = 0.020) but not tMIP-ASPECT score (OR = 1.073, 95% CI = 0.820–1.405, *p* = 0.607) was an independent predictor for recanalization after adjusting for NIHSS score and the site of occlusion (Table 2). ROC analysis also revealed that ATD predicted the occurrence of recanalization (area under curve = 0.69, 95% CI, 0.558–0.815, *p* = 0.009), with the optimal threshold of 2.30 seconds for identifying recanalization (sensitivity of 63.9% and a specificity of 70%, Youden index = 33.9%).

**Figure 3:**
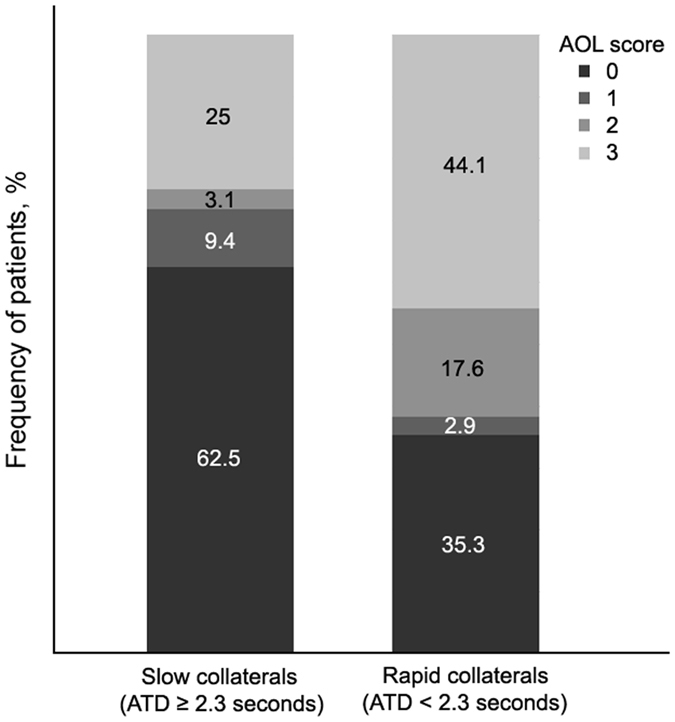
Degree of recanalization depending on the velocity of collaterals.

Patients were then dichotomized into rapid collaterals (ATD < 2.3 seconds, n = 34) versus slow collaterals (ATD ≥ 2.3 seconds, n = 32). Patients with slow collaterals had more occlusion of ICA and MCA-M1 (50% vs 23.5%, χ^2^ = 4.992, *p* = 0.025), larger baseline hypoperfusion (median: 40.99 mL vs 6.45 mL, Z = −2.773, *p* = 0.006) and infarct volume (median: 129.63 mL vs 82.73 mL, Z = −2.836, *p* = 0.005), and lower rate of recanalization(28.1% vs 61.8%, χ^2^ = 7.524, *p* = 0.006), in comparison of rapid collaterals group. The frequency of patients for AOL score regarding with collateral velocity was presented in Fig. 3.

**Figure 4:**
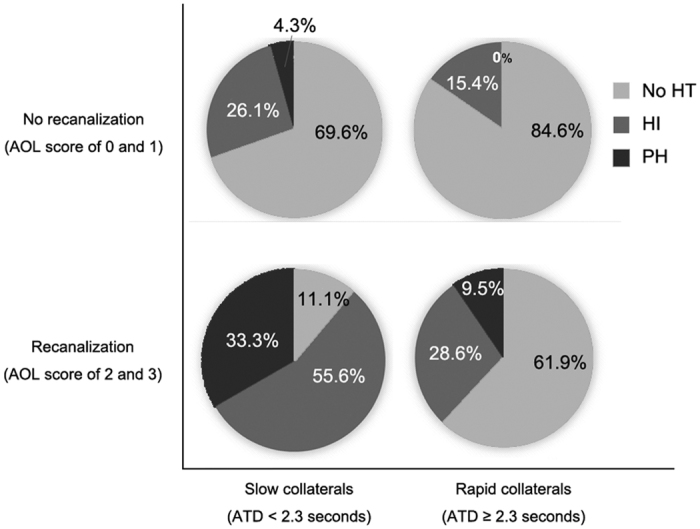
Relationship between categories of hemorrhagic transformation (HT) with the velocity of collaterals (x-axis) and the degree of recanalization (y-axis).

Higher rate of HT was detected in patients with slow collaterals than those with rapid collaterals (28.4% vs 50%, χ^2^ = 2.927, *p* = 0.087). Moreover, in patients who achieved recanalization, those with slow collaterals were more likely to have HT (88.9% vs 38.1%, χ^2^ = 6.877, *p* = 0.011), compared with rapid collaterals group. However, such difference was not observed in those without recanalization (15.4% vs 34.8%, χ^2^ = 1.588, *p* = 0.270) (Fig. 4). Importantly, all sHT (2/9) were observed in slow collaterals patients with subsequent recanalization.

## Discussion

Our study firstly demonstrated that the velocity of collateral filling was independently associated with the rate of recanalization after IVT. Patients with slow collateral filling on MRP had lower rate of recanalization, and was faced with relatively high risk of HT when recanalization was achieved after IVT. This collateral status evaluated on MRP may help the therapeutic decision-making in patients with large artery occlusion when recanalization is considered.

Our study paralleled and extended prior reports about the temporal aspect of back-filling collaterals. The association between the velocity of collaterals and recanalization can be explained as follows. First, collateral flow velocity is a surrogate for changes in the pressure drop across the collateral vessels in response to the occlusion^14^. Second, it also reflects the peripheral vascular resistance to the cerebral blood inflow through LMCs pathways within the ischemic regions^15^,^16^. Therefore, rapid collateral flow, as opposed to slow ones, may apply higher shear stress to the thrombus to cause the disruption of the clot. In addition, our data showed that patients with rapid collaterals had less clots cross ICA and MCA M1 segment than those with slow collaterals, indicating rapid collaterals were likely to be the consequence of low clot extent. The smaller clot extent, the more likely thrombus dissolved by rt-PA^17^.

Recanalization was often achieved at the cost of increased rate of HT. In this study, patients with rapid collateral filling had lower risk of HT after recanalization. For those patients, fast transfer to the angiography suit for ancillary endovascular therapy is suggested in cases of IVT failure. In contrast, patients with slow collaterals were susceptible to severe HT after recanalization. Therefore, for those patients with slow collaterals, caution may be exercised when aggressive recanalization therapy is considered. Slow blood inflow through LMCs may be associated with the stagnant outflow through cerebral venous system, resulting in the accumulation of harmful metabolites, such as active oxygen, to the vessels within ischemic regions and subsequent vascular injury^18^. Moreover, our data also showed the association between the velocity of collaterals and baseline hypoperfusion and infarct volume, supporting that slow collateral blood supply may enlarge the ischemic brain tissue as well as the injury of vessels therein.

Our finding is interesting that extensive collateral flow may not increase recanalization rate after IVT in ICA/MCA occlusion. We have to clarify that the use of ASPECTS score in this study was based on PWI raw data, which was different from the previous ASPECT score applied in PWI, including MTT-ASPECTS and CBV-ASPECTS, which was found to be associated with improved IVT efficacy in previous study. Indeed, MTT or CBV indicates the perfusion status of ischemic tissue, while hypoperfusion volumes were found to predict infarct size in SWIFT PRIME in most recent study^19^. However, our measurement of tMIP-ASPECTS derived from raw data directly reflects the extent of collateral flow. Therefore, our image mode used for the measurement of ASPECTS was different from previous ones. Two possible mechanisms may explain our finding why we did not find the association between tMIP-ASPECT score and recanalization. First, rt-PA may not work at full capacity to burst occlusion among patients with extensive collateral routes, if the functional capacity of LMCs is poor, which is the extreme case in Moyamoya vessels. Second, extensive LMCs do not necessarily re-establish arterial blood flows, but may serve as the pathways for venous flow diversion, which was known as cerebral venous steal due to the increased inflow resistance and focal compression after infarct^20^. In this case, the collateral flow may be diverted to veins, resulting in decreased effect of rt-PA on the distal end of clot.

This is the first MRP-based assessment to individually show the predictive value of collateral filling velocity and extent. Compared with previous assessments to collateral status, our methods have several strengths. Menon *et al*. employed dynamic CTA to set the time interval of retrograde filling less than 5 seconds from para-sagittal to Sylvian Sulcus as fast collateral filling^21^. Compared with this method, our ATD was normalized to contralateral delay, negating the factors such as the contrast injection and cardiac output. The other CTP-based study used the delay of time to peak (TTP) of the M2 segment of MCA as a surrogate marker for the backfilling velocity in acute ischemic stroke^9^. However, TTP that reflects the time from the beginning to maximum collateral blood flow may depend on the amount or extent of collaterals. Our ATD diminished such morphologic impact and further objectively reflected the real time that collateral blood flow acted on the thrombus. Besides, the assessment of ATD are also reproducible and fast because of the simple and programmed post-processing by MIStar software. Finally, rating ASPECT score on temporally fused images combined the visual assessment and semi-quantitative way to depict the total extent of collateral vessels, which is more intuitive and reliable to gauge the morphology of collaterals in comparison of previous methods.

Limitations in this study include a retrospective design and a potential risk of selection bias. However, patients were included in a prospective stroke registry from our stroke center with highly homogenous and standardized medical care. Second, TOF-MRA may overestimate the degree of vessel occlusion. This bias was minimized by using the movie player tool of MIStar software to exclude the patients with obvious antegrade flow beyond the clots. Third, we did not have DSA imaging in our patients for comparison. Nevertheless, MRP imaging may be superior to the former technique owing to its ability to visualize anterior and posterior circulation simultaneously, as well as to provide parenchymal information. Confirmation and extension in larger and multicenter cohorts is needed.

In conclusion, our study identified the effect of the velocity of collateral filling on recanalization after IVT. Slow collaterals may be indicative of no recanalization, and high risk of HT even if recanalization is achieved. This novel MRP imaging-based approach adds functional information of collateralization in patient selection for maximizing the beneficial effect of revascularization therapy. Further studies are warranted to validate our findings in large multicenter cohorts.

## Additional Information

**How to cite this article**: Zhang, S. *et al*. The velocity of collateral filling predicts recanalization in acute ischemic stroke after intravenous thrombolysis. *Sci. Rep.* **6**, 27880; doi: 10.1038/srep27880 (2016).

## Figures and Tables

**Table 1:** Univariate comparison between patients with or without recanalization.

	Non-recanalization (n = 36)	Recanalization (n = 30)	Test value	*P*value
Age, y	68.83 ± 13.02	69.73 ± 13.34	t = 0.765	0.783
Female, n (%)	15 (41.7)	13 (43.3)	χ^2^ = 0.019	0.891
Risk factors				
Hypertension, n (%)	26 (72.2)	21 (70.0)	χ^2^ = 0.039	0.843
Diabetes Mellitus, n (%)	9 (25.0)	9 (30.0)	χ^2^ = 0.206	0.650
Atrial fibrillation, n (%)	14 (38.9)	15 (50.0)	χ^2^ = 0.820	0.365
Hyperlipidemia, n (%)	16 (44.4)	11 (36.7)	χ^2^ = 0.409	0.522
Smoking, n (%)	13 (36.1)	11 (36.7)	χ^2^ = 0.002	0.963
Previous stroke/ TIA, n (%)	8 (22.2)	4 (13.3)	χ^2^ = 0.869	0.351
Baseline SBP, mmHg	152.1 ± 22.86	151.8 ± 20.90	t = 0.463	0.959
Baseline DBP, mmHg	82.61 ± 15.18	85.33 ± 14.0	t = 0.540	0.455
Baseline glucose, mmol/L	8.09 ± 2.54	7.77 ± 2.06	t = 0.172	0.583
Occlusion of ICA and MCA-M1, %	17 (47.2)	7 (23.3)	χ^2^ = 4.036	0.045
ATD, seconds	4.13 ± 4.88	1.67 ± 2.29	Z = −2.653	0.008
tMIP-ASPECT score	5.61 ± 2.68	6.63 ± 1.59	Z = −1.484	0.138
Onset to imaging time, OIT, minutes	196.21 ± 92.4	207.73 ± 93.75	t = 0.913	0.638
Baseline NIHSS	15 (9–18)	13.5 (5.8–16.3)	t = 0.695	0.280
Baseline infarct volume, mL	23.6 (2.93–74.98)	11.9 (4.2–35.23)	Z = −0.911	0.362
Baseline hypoperfusion volume, mL	111.1 (66.28–159.86)	102.82 (69.1–13.29)	t = 0.151	0.510

TIA, transient ischemic attack; SBP, systolic blood pressure; DBP, diastolic blood pressure; ICA, internal carotid artery; MCA-M1, M1 segment of middle cerebral artery; ATD, arrival time delay; tMIP, temporally fused maximum intensity projections; ASPECT score, Alberta Stroke Program Early CT score; OIT, onset to imaging time; NIHSS, national institute of health stroke scale.

**Table 2:** Multivariate regression analysis for recanalization at 24 hours after IVT.

	Model 1			Model 2		
	OR	95% CI	*P*value	OR	95% CI	*P*value
Baseline NIHSS score	1.006	0.911–1.110	0.871	1.189	0.935–1.512	0.158
occlusion of ICA and MCA-M1	0.437	0.141–1.353	0.151	0.340	0.117–0.991	0.048
ATD	0.775	0.626–0.960	0.020	–	–	–
tMIP-ASPECT score	1.073	0.820–1.405	0.607	1.199	0.914–1.574	0.191

IVT, intravenous thrombolysis; NIHSS, national institute of health stroke scale; ICA, internal carotid artery; MCA-M1, M1 segment of middle cerebral artery; ATD, arrival time delay; tMIP, temporally fused maximum intensity projections; ASPECT score, Alberta Stroke Program Early CT score.
